# Evaluation of workplace infection prevention and control measures for COVID-19: A prospective cohort study in Japan

**DOI:** 10.1016/j.heliyon.2023.e15996

**Published:** 2023-05-03

**Authors:** Kazushirou Kurogi, Kazunori Ikegami, Hajime Ando, Ayako Hino, Mayumi Tsuji, Yu Igarashi, Tomohisa Nagata, Keiji Muramatsu, Yoshihisa Fujino

**Affiliations:** a Department of Work Systems and Health, Institute of Industrial Ecological Sciences, University of Occupational and Environmental Health, Japan, 1-1, Iseigaoka, Yahatanishi-ku Kitakyushu-shi, Fukuoka, 807-8555, Japan; b Department of Mental Health, Institute of Industrial Ecological Sciences, University of Occupational and Environmental Health, Japan, 1-1, Iseigaoka, Yahatanishi-ku Kitakyushu-shi, Fukuoka, 807-8555, Japan; c Department of Environmental Health, School of Medicine, University of Occupational and Environmental Health, Japan, 1-1, Iseigaoka, Yahatanishi-ku Kitakyushu-shi, Fukuoka, 807-8555, Japan; d Disaster Occupational Health Center, Institute of Industrial Ecological Sciences, University of Occupational and Environmental Health, Japan, 1-1, Iseigaoka, Yahatanishi-ku Kitakyushu-shi, Fukuoka, 807-8555, Japan; e Department of Occupational Health Practice and Management, Institute of Industrial Ecological Sciences, University of Occupational and Environmental Health, Japan, 1-1, Iseigaoka, Yahatanishi-ku Kitakyushu-shi, Fukuoka, 807-8555, Japan; f Department of Public Health, School of Medicine, University of Occupational and Environmental Health, Japan, 1-1, Iseigaoka, Yahatanishi-ku Kitakyushu-shi, Fukuoka, 807-8555, Japan; g Department of Environmental Epidemiology, Institute of Industrial Ecological Sciences, University of Occupational and Environmental Health, Japan, 1-1, Iseigaoka, Yahatanishi-ku Kitakyushu-shi, Fukuoka, 807-8555, Japan

**Keywords:** Infection prevention, Infection control, COVID-19 infection, Prospective cohort study, Workplace evaluation

## Abstract

**Background:**

Encouraging the implementation of infection prevention and control (IPC) measures has been necessary to prevent workplace infections caused by the coronavirus disease 2019 (COVID-19). However, the effectiveness of these measures in reducing infections has not been thoroughly evaluated. We evaluated employees’ COVID-19 infection rates in relation to the implementation of IPC measures at their workplaces to identify effective workplace measures.

**Methods:**

This prospective cohort study was conducted between December 2020 and December 2021 using Internet-based self-assessment questionnaires, with 11,982 participants included from the baseline. To estimate whether implementing workplace IPC measures was associated with COVID-19 incidence rates among participants, we estimated multivariate-adjusted relative risk (RR) using a log-binomial model.

**Results:**

After adjusting for sex, age, education, household members, occupation-related factors, and personal preventive behaviors, requesting ill employees to refrain from going to work showed significantly lower COVID-19 infection rates than not requesting it (RR: 0.56, 95% CI: 0.34–0.91, *p* = 0.019).

**Conclusions:**

Employees restricted from reporting to work when ill had significantly lower COVID-19 infection rates than those who did not follow this measure. The results indicated that not coming to work when ill was effective in reducing COVID-19 infections at the workplace. We suggest that companies proactively adopt this policy and encourage their employees to comply with it.

## Highlights

• COVID-19 infection assessment in workplace infection prevention and control (IPC).
• This prospective cohort study was conducted from December 2020 to December 2021.
• Employees restricted from working when ill had significantly lower infection rates.
• Workplaces should prioritize implementing this measure as a workplace IPC.

## 1 Introduction

Coronavirus disease 2019 (COVID-19), caused by the severe acute respiratory syndrome coronavirus 2 (SARS-CoV-2) outbreak in December 2019, has been a global catastrophe [1,2]. As of January 1, 2023, more than 656 million confirmed cases and 6.6 million deaths have been reported worldwide [3].

One reason for the failure to prevent the spread of COVID-19 was the SARS-CoV-2 variants of concern (VOC). Five VOC have been confirmed since the onset of the pandemic through January 2022, and infections have continued to spread [4]. In Japan alone, three waves of the COVID-19 pandemic (third wave [November 2020–February 2021], fourth wave [April–June 2021], and fifth wave [July–September 2021]) were recorded in just one year (2021) [5]. Although no mandatory lockdown has been implemented in Japan, the government has repeatedly issued state (or semi-state) of emergency COVID-19 measures. Many companies followed the government's request for self-imposed restrictions and made efforts to prevent infection by curtailing economic activities, such as through temporary closures, business restrictions, and telecommuting [5]. However, with the resumption of economic activities and the recurrence of the highly infectious SARS-CoV-2 VOCs, the number of infected people has increased with each wave.

Health and safety in the workplace should be ensured during the COVID-19 pandemic because employment supports the economy and the health of nations, communities, and individuals [6]. The World Health Organization (WHO) has issued guidance outlining key preparedness, readiness, and response actions for COVID-19 and urged countries to take immediate action [6,7]. Lockdown measures were implemented in many countries to prevent the spread of COVID-19, but these had the adverse effect of halting economic activity [8]. Infection prevention measures (IPC) in the workplace were considered critical in controlling the COVID-19 pandemic while allowing economic activity to continue.

Public health (or non-pharmaceutical) interventions aimed at reducing the risk of transmission of COVID-19 have been shown to be effective in fighting respiratory infections transmitted via contact, droplet, and aerosol routes of infection [9,10]. The spread of infections must be especially controlled in the workplace, where many employees spend a substantial amount of time together daily. To prevent workplace infections, actively promoting telework and implementing infection prevention and control (IPC) measures in relation to the “3Cs” (confined spaces, crowded places, and close contact) perspective are crucial [11]. The International Labour Organization (ILO) has also presented an action checklist for infection risk reduction [12], and Japanese companies have often implemented workplace IPC measures provided in COVID-19 guidelines issued by national or academic organizations [13].

Although large companies implement these IPC measures, the rate of implementation in small and medium-sized companies is low [6]. Similarly, as found in our previous study, certain workplaces have not implemented all workplace IPC measures because of their small size, inability to afford implementing these measures, or difficulties in dealing with the situation due to the industry type [14]. While many workplaces have implemented various IPC measures, the effectiveness of these measures in curbing infections is unclear and has not been adequately evaluated.

We considered it necessary to determine the extent to which workplace IPC measures may contribute to reducing COVID-19 infections, as this could help identify priority measures to ensure the implementation of effective IPC measures in the workplace. Focusing on workplace-led IPC measures and examining their effectiveness will likely prove useful in developing more effective workplace guidelines and in dealing with future exposure to new infectious disease threats.

We conducted a prospective cohort study, called the Collaborative Online Research on Novel-coronavirus and Work study (CORoNaWork study), using data collected via online survey by the research group comprising the University of Occupational and Environmental Health, to determine the health impact of the COVID-19 pandemic on Japanese workers starting in 2020. In this study, we evaluated participants’ COVID-19 infection rates in relation to the implementation of IPC measures at each workplace using one-year prospective data collected through surveys.

## 2 Materials and methods

### 2.1 Study design

**Fig. 1:**
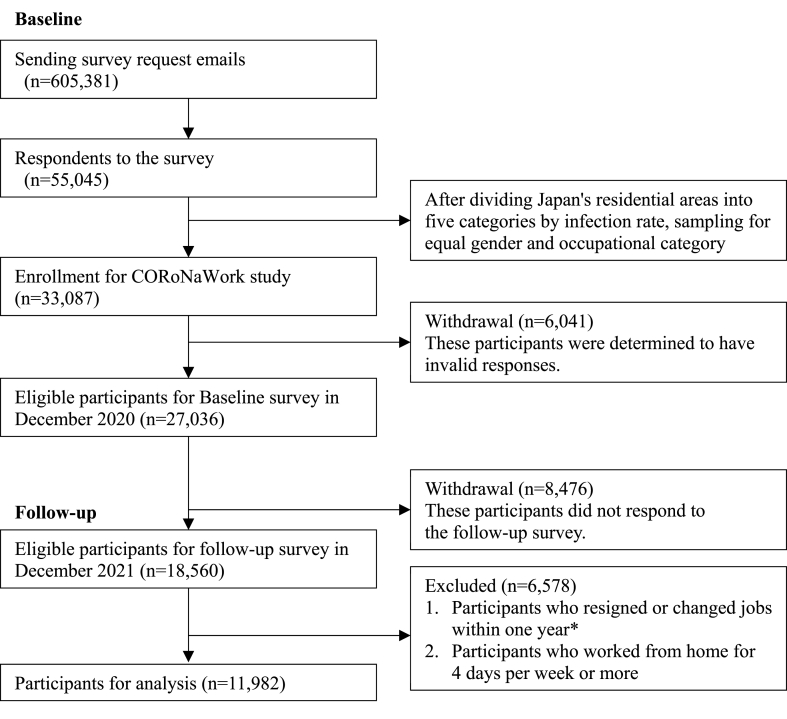
Flow chart of the sample selection procedure. * Transferred to another company, resigned and immediately took a new job, resigned and was unemployed for a period of time but now I have a job, resigned and started my own business (company management, sole proprietorship, self-employment, etc.) after December 2020.

We conducted a prospective cohort research study between December 2020 and December 2021. Baseline and follow-up surveys were conducted using Internet-based self-report questionnaires. All participants were informed about the requirements and purpose of the study, and their participation was presumed to indicate consent. The protocol for the CORoNaWork study, which included the sampling plan and subject recruitment procedures, was performed according to the CHERRIES checklist [15,16]. The participants were balanced according to the sampling plan by geographic region, sex, and type of work [17]. Details of the protocol are shown in Fig. 1.

The baseline study, initiated in December 2020, when Japan was on high alert during the third wave, showed that the number of COVID-19 cases and associated deaths was significantly higher than that of the first and second waves. In the December 2021 follow-up study, the fifth wave reached a plateau, and the incidence of infections decreased dramatically.

Participants included working adults aged 20–65 years, who were classified according to sex, region, and occupation. Regions were divided into five levels in 47 prefectures, depending on the level of infection, while occupations were divided into office workers and non-office workers, which resulted in 20 sample blocks (two sexes, five regions, and two occupations). We aimed to reach 30,000 people; hence, we sought to recruit at least 1500 individuals in each block.

This survey was commissioned by an Internet-based research provider. Among the already registered 4.7 million individuals, approximately 600,000 received e-mails asking them to participate in the survey; 55,045 of them participated in the primary screening survey, and 33,087 met the criteria for inclusion. From the 33,087 participants, 27,036 were analyzed after invalid or fraudulent responses were excluded. Exclusion criteria were as follows: brief response time (≤6 min), low body weight (<30 kg), and short height (<140 cm). Inconsistent answers to the same questions throughout the survey (e.g., answers to questions on marital status and living area being inconsistent), and incorrect answers to specific questions (e.g., “choose the third-largest number of the following five”) were used to identify fraudulent respondents. There were no participants with missing values as the survey was conducted via the constructed Internet questionnaire system.

One year after the baseline study, a follow-up study was conducted in which 18,560 (68.7%) responses were received. Among these, 6578 participants were excluded after resigning, changing jobs, or working more than four days a week at home during the study period. Regarding job change and retirement, only participants who responded “no” to the question “Have you changed jobs or retired since December 2020?” were included in the sample. Those who had changed jobs or retired (transferred to another company, resigned and immediately took a new job, resigned and was unemployed for a period of time but now I have a job, resigned and started my own business [company management, sole proprietorship, self-employment, etc.]) were excluded. This was done to prevent changes in IPC measures due to changes in the company that employed them. Regarding the frequency of telecommuting, the baseline survey asked, “Do you work from home?” The respondents were asked to select either four or more days a week, two or more days a week, one or more days a week, one or more days a month, or almost never. Those who worked from home more than 4 days a week were excluded because they were considered to be less influenced by the company's IPC measures. The final analysis included 11,982 participants (44.3%) (see Fig. 1 below).

### 2.2 Evaluation of workplace IPC measures

The participants were asked to respond with “yes” or “no” to whether the following eight COVID-19 measures were practiced in their workplaces at baseline: (1) restricting or ceasing business trips; (2) arranging health screenings for visitors; (3) restricting or ceasing work-related social gatherings and entertainment, and limiting attendees; (4) refraining from or limiting face-to-face internal meetings; (5) encouraging the wearing of masks at all times during working hours; (6) installing partitions or changing the working environment; (7) recommending daily temperature checks; and (8) requesting that employees refrain from going to work when ill.

### 2.3 Outcome and measures

COVID-19 infection after January 2021 was the outcome variable. We asked the question, “Have you been diagnosed with COVID-19 since January 2021?” We defined those who answered “yes” as a “COVID-19 infected person” during the follow-up period. Whether each of the eight previously defined workplace IPC measures were implemented at baseline was the predictor variable*.*

As confounding variables, we used the following items surveyed at baseline: sex, age (20–29, 30–39, 40–49, 50–59, or ≥60 years), education (junior high/high school, vocational school/college, or university/graduate school), number of household members (one, two, three, or ≥ four people), standard industrial classification (primary, secondary, and tertiary), job type (regular employees, managers, or other), and size of workplace (1–9, 10–49, 50–999, or ≥1000 people). Additionally, we considered six personal preventive behaviors as confounding variables. Participants answered questions on these personal preventive behaviors using a 4-point scale, with answers as follows: “almost always,” “often,” “not often,” and “almost never.” Each answer was treated as a binary variable in terms of being either “almost always” or “other” for the other three responses. The questions were related to: (1) wearing a mask in the presence of others; (2) disinfecting hands with alcohol; (3) gargling on returning home; (4) opening windows and doors to ventilate the room; (5) carrying alcohol disinfectant when going out; and (6) disinfecting and washing hands after touching things that many people have touched.

### 2.4 Statistical methods

To estimate whether IPC measures were related to the incidence rates of COVID-19 among the participants, multivariate-adjusted relative risk (RR) was estimated using a log-binomial model. The model included age, sex, education, number of household members, standard industrial classification, job type, size of workplace, personal preventive behaviors, and the eight COVID-19 infection control measures as the explanatory variables. All models included the presence or absence of implementing each IPC as an explanatory variable. Model 1 was adjusted for sex and age; Model 2 for sex, age, education, number of household members, and occupation-related factors, such as standard industrial classification, job type, and size of workplace; and Model 3 for personal preventive behaviors. In all tests, the threshold for significance was set at *p* < 0.05. Stata/SE Ver.17.0 (StataCorp LLC, College Station, TX) software was used for the analysis.

## 3 Results

Table 1 shows the participants’ baseline characteristics. Among the 11,982 participants, the number (proportion) of male participants was higher (56.8%) than female participants (43.2%), and the majority (37.1%) of participants were aged 50–59 years. Most participants had university- or graduate school-level education. Regarding occupation-related factors, tertiary industry, which had the highest percentage of employees (72.8%), and workplaces with 50–999 employees (34.2%), had the highest representation. Among personal preventive behaviors for COVID-19 infection, wearing a mask in the presence of others was the most frequent behavior (85.6%) among the participant groups, followed by disinfecting hands with alcohol (53.4%), and gargling on returning home (51.1%).

**Table 1:** Participants’ baseline characteristics.

Items	N	(%)
Total participants	11982	(100.0)
Sex		
Male	6800	(56.8)
Female	5182	(43.2)
Age		
20–29 years	579	(4.8)
30–39 years	1880	(15.7)
40–49 years	3832	(32.0)
50–59 years	4445	(37.1)
≥60 years	1246	(10.4)
Education		
Junior high or High school	3332	(27.8)
Vocational school or College	2682	(22.4)
University or Graduate school	5968	(49.8)
Number of household members		
One person	2325	(19.4)
Two people	3204	(26.7)
Three people	3066	(25.6)
≥Four people	3387	(28.3)
Standard industrial classification†		
Primary industry	111	(0.9)
Secondary industry	3150	(26.3)
Tertiary industry	8721	(72.8)
Job type		
Regular employees	5645	(47.1)
Managers	1348	(11.3)
Other	4989	(41.6)
Size of workplace		
1–9	3295	(27.5)
10–49	3494	(29.2)
50–999	4097	(34.2)
≥1000	1096	(9.1)
Personal preventive behaviors for COVID-19 infection‡		
Wearing a mask in the presence of others	10252	(85.6)
Disinfecting hands with alcohol	6396	(53.4)
Gargling on returning home	6121	(51.1)
Opening windows and doors to ventilate the room	5242	(43.8)
Carrying alcohol disinfectant when going out	3439	(28.7)
Disinfecting hands and washing hands after touching things that many people have touched	4146	(34.6)

†Mining, quarrying, and gravel extraction are classified as secondary industries.

‡Number of “Almost always” responses are indicated here.

Table 2 shows workplace IPC measures and the number of COVID-19 infections. Among all the workplace IPC measures, “encouraging the wearing of masks at all times during working hours” had the highest implementation rate (79.9%), followed by “requesting that employees refrain from going to work when ill” (76.2%) and “restricting work-related social gatherings and entertainment” (71.9%). When the COVID-19 infection rate corresponding to each of the measures was considered, “requesting that employees refrain from going to work when ill” (1.00%) had the lowest rate, followed by “restricting work-related social gatherings and entertainment” (1.02%).

**Table 2:** Workplace IPC measures and the number of COVID-19 infections.

Implementation status of workplace IPC measures		n	# of COVID-19 infections	
			n	(%)
Restricting or ceasing business trips	Yes	6554	73	(1.11)
	No	5428	64	(1.18)
Arranging health screenings for visitors	Yes	5331	65	(1.22)
	No	6651	72	(1.08)
Restricting work related social gatherings and entertainment	Yes	8618	88	(1.02)
	No	3364	49	(1.46)
Restricting face to‐face meetings	Yes	6474	76	(1.17)
	No	5508	61	(1.11)
Encouraging mask-wearing at work	Yes	9568	104	(1.09)
	No	2414	33	(1.37)
Installing partitions or changing the working environment	Yes	7120	78	(1.10)
	No	4862	59	(1.21)
Enforcing temperature measurement	Yes	7737	88	(1.14)
	No	4245	49	(1.15)
Requesting ill employees to refrain from going to work	Yes	9131	91	(1.00)
	No	2851	46	(1.61)

IPC: infection prevention and control.

Table 3 shows the association between the implementation of each measure and COVID-19 infection. In the model adjusted for sex and age (Model 1), requesting that ill employees refrain from going to work resulted in a significantly lower COVID-19 infection rate RR than not requesting the same (RR: 0.54; 95% confidence interval [CI]: 0.33–0.87, p = 0.012). The RR was also significantly lower for Model 2, which included education, household members, and occupation-related factors (RR: 0.55; 95% CI: 0.34–0.90, p = 0.017). Model 3, which included personal preventive behaviors, had an RR of 0.56 (95% CI: 0.34–0.91, p = 0.019). This measure had significantly lower RR among all models. “Restricting work-related social gatherings and entertainment” had a marginally significant lower RR for COVID-19 infection compared to not restricting the same (RR: 0.59; 95% CI: 0.35–1.02, p = 0.057). Marginally significant trends were observed in Model 2 (RR: 0.61; 95% CI: 0.35–1.05, p = 0.072) and Model 3 (RR: 0.61; 95% CI: 0.35–1.05, p = 0.072). For the other six measures, we observed no significant difference in COVID-19 infections with respect to implementation and non-implementation across all models.

**Table 3:** Association between implemented workplace IPC measures and COVID-19 infections.

Implementation status of workplace IPC measures		Model 1			Model 2			Model 3		
		RR	[95%CI]	p	RR	[95%CI]	p	RR	[95%CI]	p
Restricting or ceasing business trips	Yes	1.04	[0.64–1.71]	0.864	1.03	[0.62–1.70]	0.923	1.02	[0.62–1.69]	0.931
	No	ref.			ref.			ref.		
Arranging health screenings for visitors	Yes	1.36	[0.86–2.15]	0.193	1.30	[0.81–2.08]	0.270	1.32	[0.83–2.11]	0.244
	No	ref.			ref.			ref.		
Restricting work‐related social gatherings and entertainment	Yes	0.59	[0.35–1.02]	0.057	0.61	[0.35–1.05]	0.072	0.61	[0.35–1.05]	0.072
	No	ref.			ref.			ref.		
Restricting face‐to‐face meetings	Yes	1.41	[0.86–2.32]	0.178	1.36	[0.82–2.25]	0.231	1.37	[0.83–2.26]	0.217
	No	ref.			ref.			ref.		
Encouraging mask wearing at work	Yes	0.99	[0.60–1.65]	0.973	1.03	[0.62–1.72]	0.904	1.04	[0.62–1.74]	0.877
	No	ref.			ref.			ref.		
Installing partitions or changing the working environment	Yes	0.99	[0.64–1.53]	0.972	0.99	[0.64–1.54]	0.959	1.00	[0.64–1.56]	0.996
	No	ref.			ref.			ref.		
Enforcing temperature measurement	Yes	1.23	[0.80–1.90]	0.351	1.25	[0.81–1.95]	0.313	1.28	[0.82–1.99]	0.272
	No	ref.			ref.			ref.		
Requesting that employees refrain from going to work when ill	Yes	0.54	[0.33–0.87]	0.012	0.55	[0.34–0.90]	0.017	0.56	[0.34–0.91]	0.019
	No	ref.			ref.			ref.		

IPC: Infection Prevention and Control; RR: Relative Risk; CI: Confidence Interval; ref: reference.

Model 1: adjusted for sex and age; Model 2: adjusted for sex, age, education, number of household members, standard industrial classification, job type, and size of the workplace; Model 3: adjusted for sex, age, education, number of household members, standard industrial classification, job type, size of the workplace, and personal preventive behaviors for COVID-19 infection.

## 4 Discussion

In this study, we evaluated the effectiveness of each of eight COVID-19 workplace IPC measures implemented from December 2020 to December 2021 in Japan. We identified that participants working in companies that restricted ill employees from going to work showed significantly lower COVID-19 infection rates than those working in workplaces without this measure. We obtained the same results after further adjusting for occupation-related factors (standard industrial classification, job type, and size of workplace), and personal preventive behaviors. Thus, we suggest that requesting ill employees to refrain from going to work could help control COVID-19 infections considerably.

During infectious disease epidemics, especially those involving respiratory and gastrointestinal infections, going to work despite feeling ill may increase the risk of passing infections to co-workers and visitors [18]. During the COVID-19 pandemic, guidelines issued by ILO, governments, and academic organizations stated that employees with a fever, common cold symptoms, or other illnesses should not go to work or attend public outings due to the possibility of spreading the COVID-19 infection [12,13]. To implement such measures effectively, various countries have enacted laws, such as mandatory self-quarantine, compensated absence, and special leave for those with infected family members, to protect infected employees or those suspected of being infected [19]. Organizations that have implemented health and safety management systems in the workplace have reported greatly enhanced preventive measures against COVID-19 [6]; using these systems to enforce policies in the workplace would be important step in preventing the spread of COVID-19 amongst employees. This study suggests that policies that encourage employees to stay away from the workplace when feeling ill may be effective in controlling COVID-19 infection, and that establishing policies in the workplace and communicating them to workers would be important in preventing the spread of infection.

This study identified that participants working in workplaces that restricted work-related social gatherings and entertainment had lower COVID-19 infection rates than those working in workplaces that did not, showing marginal significance in all models. It has been reported that COVID-19 is mainly transmitted via saliva droplets and micro-droplets discharged when coughing, sneezing, or talking loudly [20]. Cases of COVID-19 infection clusters caused by social gatherings have often been reported; eating out in large groups, being in situations where sufficient physical distancing cannot be maintained, and eating as well as drinking in poorly ventilated and crowded spaces have been identified as risk factors [21,22]. Thus, the “restricting work-related social gatherings and entertainment” measure could also effectively reduce COVID-19 infections.

Regarding the six remaining workplace IPC measures, none of the models showed significant differences in COVID-19 infection rates with and without implementation. Non-woven masks, which might be highly effective in preventing droplet emission, might not protect against exposure to droplets or micro-droplets [23]. The possibility of aerosol transmission of COVID-19 and the strong infectivity of the mutant virus have also been pointed out [1,24], suggesting that wearing masks alone may be ineffective in preventing infection. Partitions have been reported to have the potential to obstruct ventilation if improperly installed [25]. Body temperature measurement has also been reported as ineffective as about one-third of COVID-19 infected patients are asymptomatic [26], and the commonly used non-contact body temperature measuring device is affected by environmental factors, such as measurement location, temperature, and humidity, and has problems with measurement accuracy [27]. These factors may have contributed to the failure to prevent infection.

It is necessary to consider that some of the effects of these measures could be modified by personal preventive behavioral factors. For example, most of the participants in this study reported that they almost always wore masks, a behavior that could be driven by personal preventive inclinations rather than workplace directives. Measuring body temperature before coming to work also substantially depends on individual preventive behaviors. Regarding conducting business trips or handling visitors, even if the participants’ companies were able to manage these with low risk, other restrictions may have made it difficult to continue these activities. Encouraging the use of masks, installing office partitions, and restricting face-to-face meetings in the workplace could have been influenced by company size, the number of personnel, and the quality of room ventilation. For these reasons, it is difficult to identify which of these factors is likely to have been effective. As such, the effects of these IPC measures require further evaluation.

We also obtained data on vaccination status and vaccination time from the participants. However, because the time at which a participant may have been infected with COVID-19 was unknown, the effectiveness of the workplace IPC measures examined in this study may have been relatively weakened because of vaccination. However, concerns about breakthrough infections even after vaccination and the possibility of attenuated vaccine efficacy due to COVID-19 variants have been raised [28]. From this perspective, requiring ill employees to refrain from going to work, which had gained early approval as an effective IPC against COVID-19, can be considered a measure that should be maintained.

This study has some limitations. First, this survey was conducted on the Internet. Only those who agreed and responded were included in the survey. This study's findings should be generalized with caution as the participants may not represent the general population. For example, there is a risk of overestimation if multiple participants were employed at the same company. To address such possible effects, we reduced selection bias through random sampling stratified by sex and region. Second, as we obtained information from the participants about the implementation of workplace IPC measures, this information may have been subject to participant bias, even if the companies were actively implementing these measures. Relevant guidelines also recommend many IPC measures other than those investigated in this study, such as improved ventilation performance and employee education on COVID-19 [13,19]. Therefore, future research should evaluate a broader scope of COVID-19 workplace IPC measures using more accurate information from occupational health staff who actively manage these measures.

## 5 Conclusion

We evaluated the effectiveness of eight COVID-19 workplace IPC measures through a one-year Internet-based prospective cohort study. We found that “requesting ill employees to refrain from going to work” appeared to have the most substantial effect on reducing COVID-19 infection, followed by “restricting work-related social gatherings and entertainment.” We suggest that prioritizing the implementation of these measures will contribute significantly to reducing COVID-19 infections.

## Ethics statement

This study was approved by the Ethics Committee of the University of Occupational and Environmental Health, Japan (reference No. R2-079 and R3-006). This study was conducted in accordance with the Declaration of Helsinki.

## Author contribution statement

Kazushirou Kurogi: Conceived and designed the experiments; Performed the experiments; Analyzed and interpreted the data; Contributed reagents, materials, analysis tools or data; Wrote the paper.

Kazunori Ikegami; Hajime Ando: Conceived and designed the experiments; Performed the experiments; Analyzed and interpreted the data; Contributed reagents, materials, analysis tools or data.

Ayako Hino; Mayumi Tsuji; Yu Igarashi; Tomohisa Nagata; Keiji Muramatsu; Yoshihisa Fujino: Conceived and designed the experiments; Performed the experiments; Contributed reagents, materials, analysis tools or data.

## Funding statement

This study was supported and partly funded by the 10.13039/501100003478Japanese Ministry of Health, Labour and Welfare (H30-josei-ippan-002, H30-roudou-ippan-007, 19JA1004, 20JA1006, 210301-1, and 20HB1004); Anshin Zaidan (no grant number), the Collabo-Health Study Group (no grant number), and Hitachi Systems, Ltd. (no grant number), and scholarship donations and a research grant from 10.13039/100016239the University of Occupational and Environmental Health, Japan (no grant number). The funders were not involved in designing the study; collecting, analyzing, and explaining data; or writing the article.

## Data availability statement

Data will be made available on request.

## Declaration of competing interest

The authors declare that they have no known competing financial interests or personal relationships that could have appeared to influence the work reported in this paper.
